# Contemporary Incidence and Procedural Volume of Transcatheter Aortic Valve Reintervention

**DOI:** 10.1001/jamacardio.2025.3224

**Published:** 2025-09-24

**Authors:** Maxwell C. Braasch, Sophia R. Pyeatte, June He, Mehran Rahimi, Alexander A. Brescia, Puja Kachroo, Harold G. Roberts, Nathan Frogge, Nishath Quader, Marc A. Sintek, Alan Zajarias, Nicholas Kouchoukos, Tsuyoshi Kaneko

**Affiliations:** 1 Division of Cardiothoracic Surgery, Department of Surgery, Washington University in St Louis, St Louis, Missouri; 2 Division of Cardiovascular Medicine, Department of Medicine, Washington University in St Louis, St Louis, Missouri

## Abstract

**Importance:**

Transcatheter aortic valve replacement (TAVR) reintervention is performed by either redo TAVR or TAVR explant. There is insufficient data on contemporary TAVR reintervention procedural incidence and volume.

**Objective:**

To evaluate contemporary procedural volumes of redo TAVR and TAVR explant.

**Design, Setting, and Participants:**

This retrospective analysis of redo TAVR and TAVR explant annual incidence and procedural volume was performed from January 2012 to June 2024. For comprehensive assessment of aortic valve replacement, analysis of SAVR reintervention annual incidence and procedural volume was also performed. Data were obtained through the US Centers for Medicare & Medicaid Services Virtual Research Data Center. Patients with prior TAVR or SAVR who underwent subsequent TAVR or SAVR were included.

**Exposures:**

Patients who underwent a TAVR after TAVR defined the redo TAVR group, while patients who underwent SAVR after TAVR defined the TAVR explant group. Patients who underwent a TAVR after SAVR defined the valve-in-valve (ViV) TAVR group, while patients who underwent SAVR after SAVR defined the redo SAVR group. Analysis of annual incidences and procedural volumes of all groups at different time intervals was conducted.

**Main Outcomes and Measures:**

The primary outcome was annual incidence and procedural volume of both redo TAVR and TAVR explant. The secondary outcome was annual incidence and procedural volume of both ViV TAVR and redo SAVR.

**Results:**

Of 410 720 total TAVRs from 2012 to 2024, 2374 redo TAVRs and 1346 TAVR explants were identified. Of 299 780 total SAVRs from 2012 to 2024, 5044 ViV-TAVRs and 4202 redo SAVRs were identified. Since 2020, there have been 1518 redo TAVRs and 1007 TAVR explants. The annual incidence of TAVR reintervention has changed from 0.17% in 2019 to 0.28% in 2023. Although the most common time interval for redo TAVR after index TAVR was within 3 months (410 of 2374 [17.3%]) and for TAVR explant after index TAVR was 1 to 2 years (259 of 1346 [19.2%]), redo TAVR was the predominant procedure beyond 5 years from the index TAVR (725 of 819 [88.5%]).

**Conclusions and Relevance:**

In this study, annual volumes of both redo TAVR and TAVR explant continued to increase, particularly in recent years. Redo TAVR is frequently performed over 5 years from the index TAVR. Future analysis to determine the appropriate TAVR reintervention strategy for individual patients is needed.

## Key Points

**Question:**

What are the contemporary procedural volumes of transcatheter aortic valve replacement (TAVR) reintervention on a national scale?

**Findings:**

In this US Centers for Medicare & Medicaid Services analysis of 410 720 TAVRs from 2012 to 2024, there were 2374 redo TAVRs and 1346 TAVR explants, demonstrating increasing annual volumes of both redo TAVR and TAVR explants. Beyond 5 years from the index TAVR, most TAVR reinterventions were redo TAVR (725 of 819 [88.5%]).

**Meaning:**

Continued focus on TAVR reintervention is critical to optimize the lifetime management of aortic stenosis.

## Teaser

This study evaluates contemporary procedural volumes of redo transcatheter aortic valve replacement and transcatheter aortic valve replacement explant.

## Introduction

Transcatheter aortic valve replacement (TAVR) is more frequently performed for aortic valve stenosis (AS) than surgical aortic valve replacement (SAVR).^1^ After the results of randomized clinical trials of low-risk patients in 2019,^2,3^ TAVR became an option for patients of all surgical risk levels. Although the American College of Cardiology/American Heart Association guidelines suggest that young, non–high-risk patients younger than 65 years should undergo SAVR,^4^ nearly 50% of younger patients in the US receive TAVRs.^5^ However, it is important to consider that younger age does not always correlate with lower procedural risk. Since all bioprosthetic valves will develop structural valve degeneration, we anticipate a rise in TAVR reintervention, especially in low-risk patients with long life expectancy.

Two procedures exist for TAVR reintervention: redo TAVR and TAVR explant. There is a paucity of data on temporal procedural incidence and volume of TAVR reintervention on a nationwide scale. The primary objective of this study is to evaluate contemporary national procedural volumes of redo TAVR and TAVR explant.

## Methods

### Study Design and Patient Selection

A retrospective analysis of US Centers for Medicare & Medicaid Services (CMS) data was performed (eFigure 1 in Supplement 1). This study was approved by the Washington University in St Louis Institutional Review Board and was deemed exempt due to the deidentified nature of the data (202301098). CMS Virtual Research Data Center data were used to access Medicare beneficiary claims with *International Classification of Diseases-9 International Classification of Diseases, Ninth Revision (ICD-9)*and *ICD-10* procedure codes indicating an index TAVR or SAVR between January 1, 2012, and June 30, 2024. Patients who underwent an index SAVR were also included for comprehensive assessment of aortic valve replacement reintervention annual incidence and procedural volume. TAVR and SAVR *ICD* procedure codes were used to identify TAVR or SAVR reinterventions in a different admission as the index TAVR or SAVR. Patient comorbidities and concomitant operations were characterized through *ICD-9* and *ICD-10* diagnosis codes.

### Group Creation and Procedural Incidence and Volume Analysis

Two TAVR reintervention groups were created: patients who underwent SAVR after prior TAVR (TAVR explant) and patients who underwent TAVR after TAVR (redo TAVR). Two SAVR reintervention groups were created: patients who underwent TAVR after prior SAVR, or valve-in-valve TAVR (ViV-TAVR), and SAVR after SAVR (redo SAVR). The volume of reintervention procedures performed annually between 2012 and 2024 was determined for all patients and for patients not diagnosed with endocarditis. The annual TAVR and SAVR reintervention incidence was calculated for all TAVRs and SAVRs at risk of reintervention each year. The time interval between the index TAVR and the TAVR reintervention was determined for redo TAVR and TAVR explant.

## Results

### Patient Characteristics

Of 410 726 patients who underwent TAVR, only 3720 patients (0.91%) underwent TAVR reintervention. Of this 3720, 2374 (63.8%) underwent redo TAVR (63.8%) and 1346 (36.2%) underwent TAVR explant (eTable 1 and eTable 2 in Supplement 1). Patients who underwent redo TAVR were an average of 80 (SD, 8) years old with 86.8% (2061 of 2374) diagnosed with congestive heart failure. Of 1346 total TAVR explants, 465 (34.5%) were performed for endocarditis. Concomitant coronary artery bypass grafting (CABG) was performed in 16.1% (217 of 1346) of TAVR explants, concomitant mitral valve surgery was performed in 25% (336 of 1346) of TAVR explants, and concomitant thoracic aortic surgery was performed in 14.9% (200 of 1346) of TAVR explants.

### Procedural Incidence and Volume

**Figure 1.**
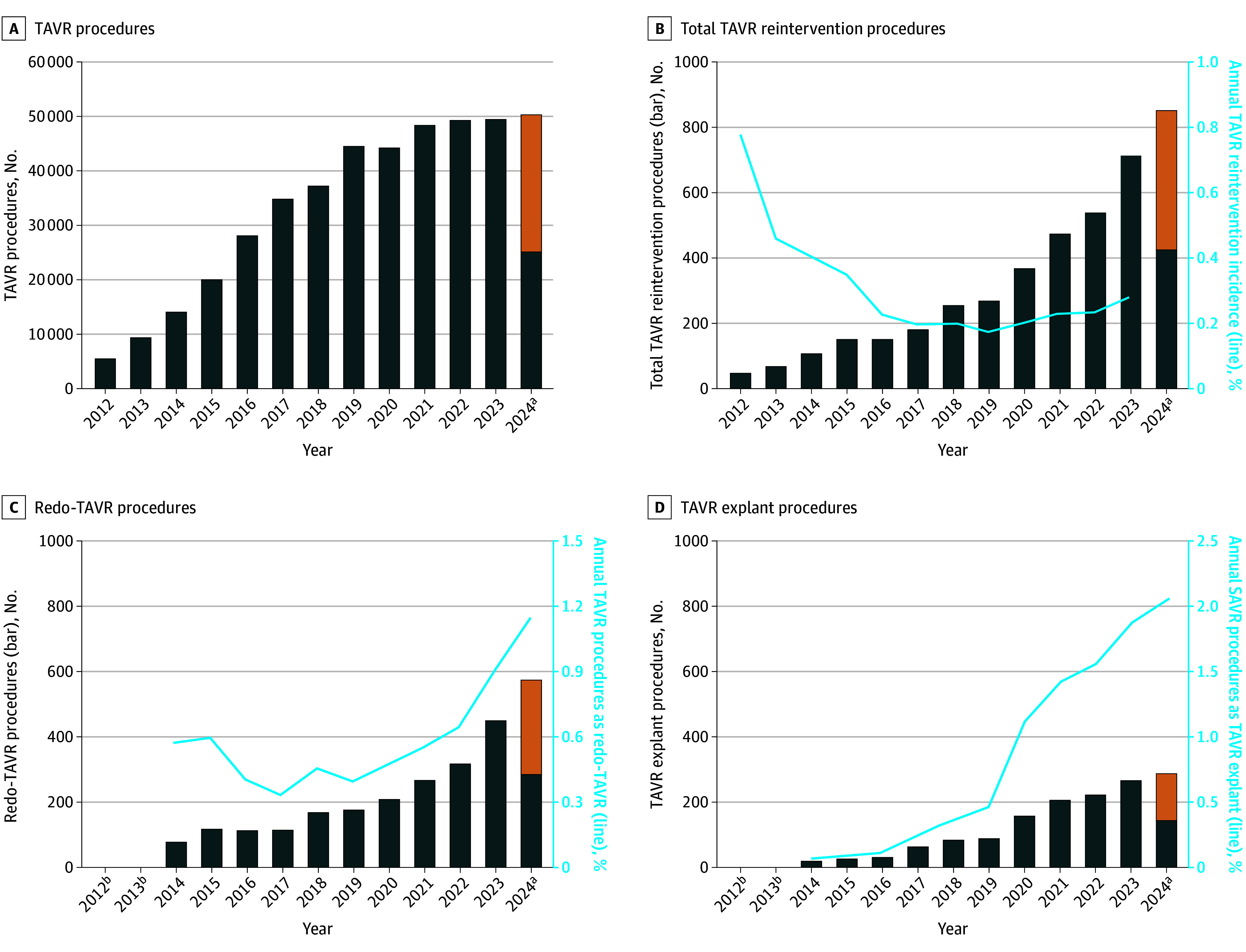
Annual Volume of Transcatheter Aortic Valve Replacement (TAVR) and TAVR Reintervention Procedures A, Number of TAVR procedures performed per year. B, Total number of TAVR reintervention procedures performed per year (bars) and the annual TAVR reintervention incidence (line). C, Number of redo TAVR procedures performed per year (bars) and the annual percentage of all TAVR procedures that are redo TAVR procedures (line). D, Number of TAVR explant procedures performed per year (bars) and the annual percentage of all surgical aortic valve replacement (SAVR) procedures that are TAVR explant procedures (line). The left y-axis scale for panel A differs from the y-axis scales for panels B through D and the right y-axis scales for panels B through D all differ from one another. ^a^Procedural volume for 2024 annualized (orange bar) from 6 months of available data (blue bar). ^b^Data censored with results <11 per Healthcare Cost & Utilization Project Data Use Agreement for patient confidentiality.

Annual procedural volume of both redo TAVR and TAVR explant are increasing, particularly in recent years, for the overall study population (Figure 1; eTable 3 in Supplement 1). The annual incidence of TAVR reintervention for the overall cohort has changed from 0.17% in 2019 to 0.28% in 2023. Similarly, annual procedural volume of both redo TAVR and TAVR explant are increasing for the nonendocarditis cohort (eFigure 2 and eTable 4 in Supplement 1).

**Figure 2.**
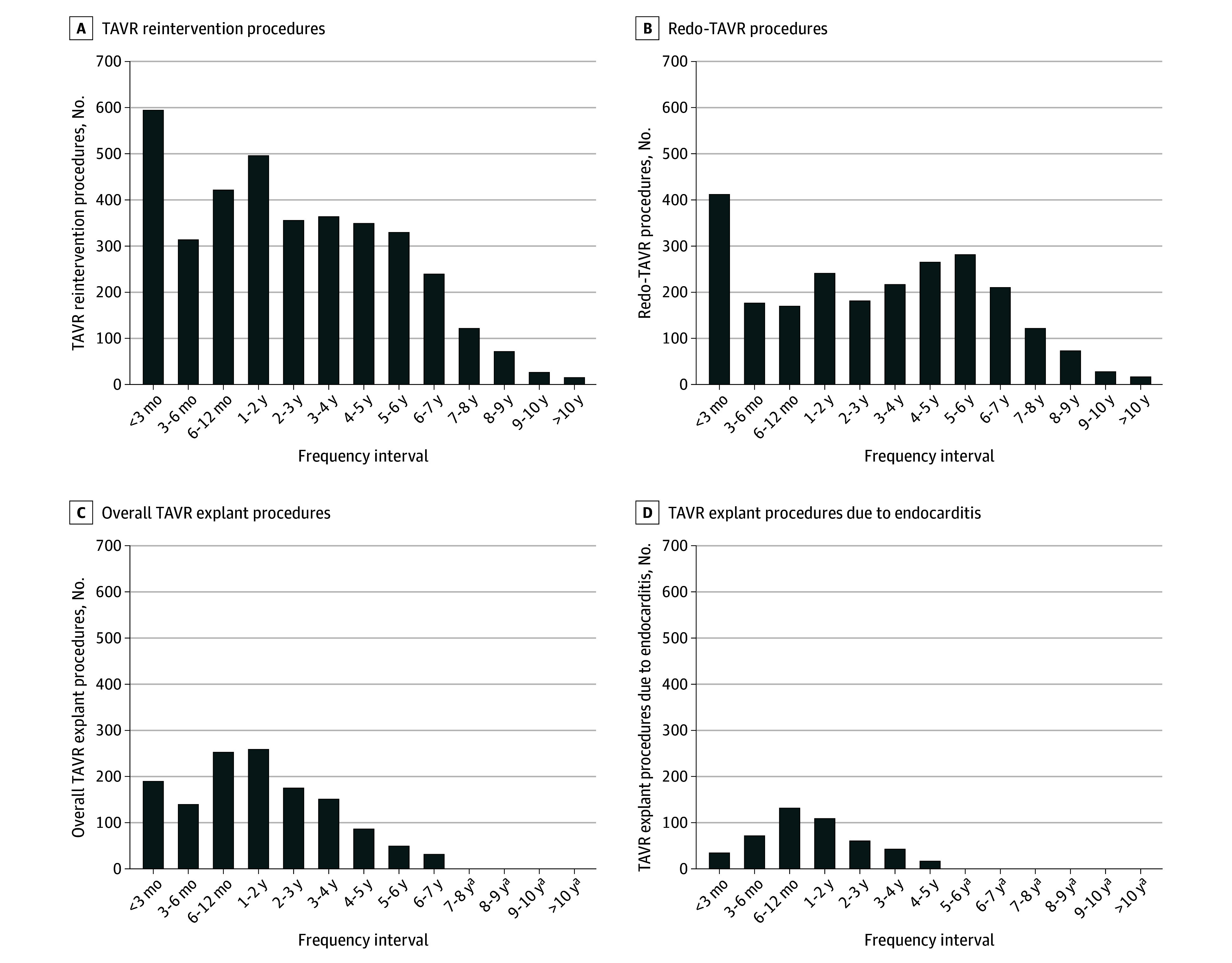
Frequency of Transcatheter Aortic Valve Replacement (TAVR) Reinterventions at Different Time Intervals From the Index TAVR A, All TAVR reinterventions at different time intervals from the index TAVR. B, Number of redo TAVRs at different time intervals from the index TAVR. C, Number of all TAVR explants at different time intervals from the index TAVR. D, Number of TAVR explants due to endocarditis at different time intervals from the index TAVR. ^a^Data censored with results <11 per Healthcare Cost & Utilization Project Data Use Agreement for patient confidentiality.

The most common time interval for redo TAVRs after the index TAVR was the first 3 months postoperatively (410 of 2374 [17.3%]), while the most common time interval for TAVR explants was between 1 and 2 years postoperatively (259 of 1346 [19.2%]) (Figure 2; eTable 5 in Supplement 1). Beyond 5 years from the index TAVR, most of the 819 TAVR reinterventions were redo TAVRs as compared with TAVR explants (725 of 819 [88.5%] vs 94 of 819 [11.5%]).

**Figure 3.**
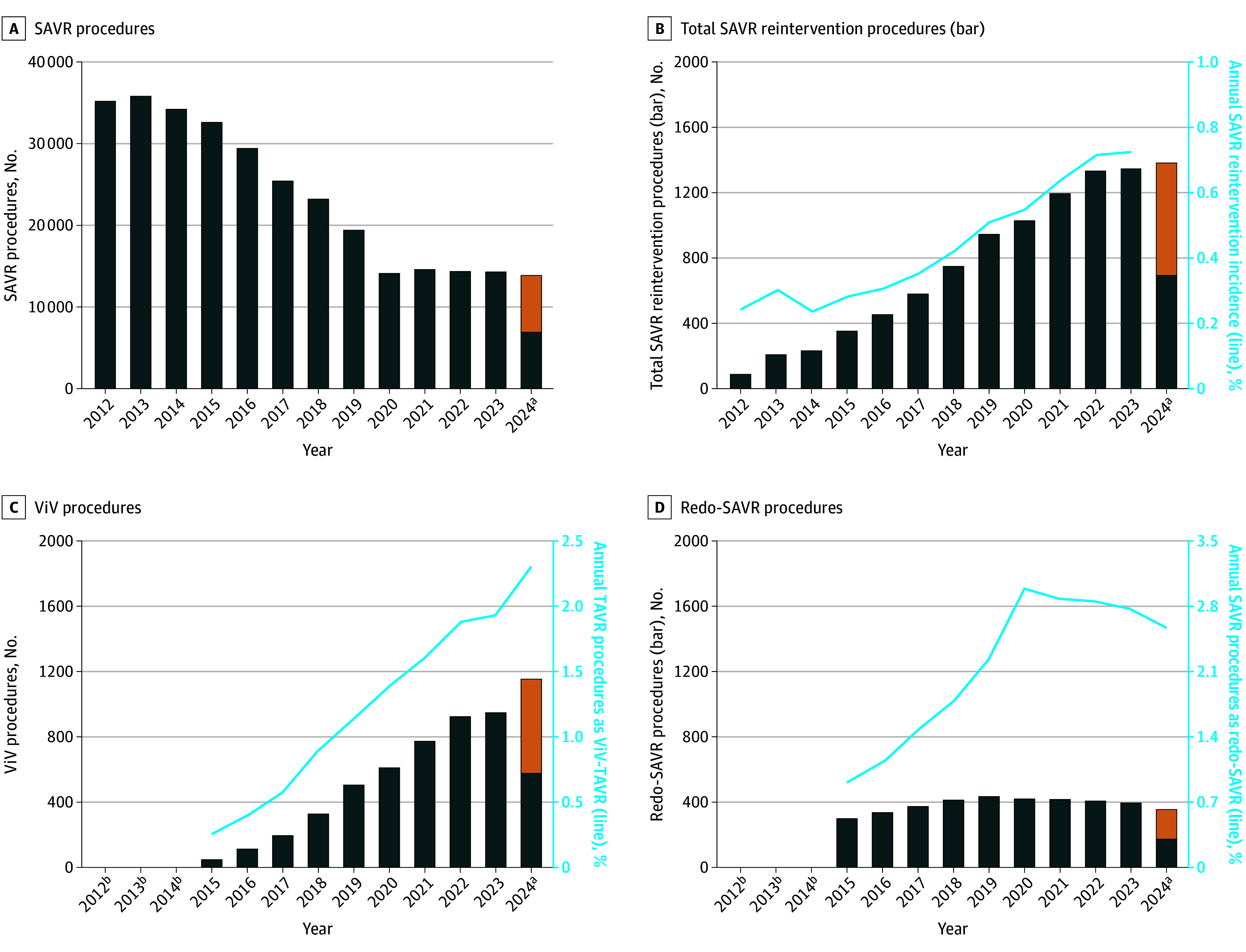
Annual Volume of Surgical Aortic Valve Replacement (SAVR) and SAVR Reintervention Procedures A, Number of SAVR procedures performed per year. B, Total number of SAVR reintervention procedures performed per year (bars) and the annual SAVR reintervention incidence (line). C, Number of valve-in-valve (ViV) transcatheter aortic valve replacement (TAVR) procedures performed per year (bars) and the annual percentage of all transcatheter aortic valve replacement procedures that are ViV-TAVR procedures (line). D, Number of redo SAVR procedures performed per year (bars) and the annual percentage of all SAVR procedures that are redo SAVR procedures (line). The left y-axis scale for panel A differs from the y-axis scales for panels B through D and the right y-axis scales for panels B through D all differ from one another. ^a^Procedural volume for 2024 annualized (orange bar) from 6 months of available data (blue bar). ^b^Data censored with results <11 per Healthcare Cost & Utilization Project Data Use Agreement for patient confidentiality.

Of 299 780 patients who underwent SAVR, 5044 (1.68%) underwent ViV TAVR and 4202 (1.40%) underwent redo SAVR (Figure 3; eTable 6 in Supplement 1). While the annual procedural volume of ViV TAVR is increasing annually with 1036 performed in 2024, the annual procedural volume of redo SAVR has decreased from 436 in 2019 to 356 in 2024. The annual incidence of SAVR reintervention for the overall cohort has changed from 0.24% in 2014 to 0.73% in 2023.

## Discussion

This study demonstrates important findings regarding TAVR reintervention. First, although the incidence of TAVR reintervention was low, the annual volume of redo TAVR, TAVR explant, and overall TAVR reintervention has increased. Second, although the highest incidence of redo TAVRs was in the first 3 months after the index TAVR, most TAVR reinterventions after 5 years were redo TAVR. These findings inform the current practice of TAVR reintervention using contemporary CMS data, 5 years after TAVR approval for low-risk groups.

Our results show an increase in annual volume of TAVR reintervention during the study period. The increase in annual volume of SAVR reintervention is driven by the increase in ViV TAVR secondary to TAVR procedural expansion. The annual TAVR reintervention incidence was <1% each year, similar to prior data.^5,6^ While the annual TAVR reintervention incidence slightly increased from 0.17% in 2019 to 0.28% in 2023, the annual SAVR reintervention incidence was higher over this same time period. It is important to continue monitoring annual TAVR and SAVR reintervention incidence and volume. We expect a continued increase in the TAVR reintervention incidence as we move to more lower risk patients, emphasizing the importance of lifetime management of AS.

Over 60% of TAVR reintervention occurs within 1 year of the index TAVR,^7^ contrary to 35.9% in our analysis. Early reintervention likely represents technical failure of the index TAVR, such as paravalvular regurgitation or patient-prosthesis mismatch. Our finding that 17.2% of redo TAVRs occur before 3 months indicates improvement in TAVR technology, implant technique, and patient selection.

TAVR explant becomes the predominant TAVR reintervention procedure between 6 months and 2 years following index TAVR. This is likely secondary to endocarditis and concomitant cardiac disease. In the EXPLANTREDO-TAVR analysis,^6^ rates of TAVR explant with concomitant CABG was 17.7% and concomitant mitral valve surgery was 20.4%, similar to our results. Interestingly, beyond 5 years after TAVR, nearly 90% of TAVR reintervention is redo TAVR. There may be selection bias that older high-risk patients requiring TAVR reintervention beyond 5 years may not have been candidates for TAVR explant. Nonetheless, prior simulations of redo TAVR feasibility estimated redo TAVR is unfeasible in nearly 40% of patients.^8^ Our results suggest that redo TAVR could become the main procedure for long-term TAVR reintervention.

Further optimization of TAVR reintervention is needed. Standardized TAVR explant operative techniques are crucial.^9^ TAVR explant may be appropriate for those with concomitant coronary/valve disease, endocarditis, and patient-prosthesis mismatch . On the other hand, patients with suitable anatomy should be considered for redo TAVR. Leaflet modification technologies, including the Basilica,^10^ the Unicorn procedure,^11^ and the dedicated device,^12^ may help mitigate coronary artery anatomic contraindications for redo TAVR. Future studies focusing on reintervention on younger, low-risk patients will be critical as the tsunami of TAVR continues to rise in this population that will undergo more interventions for AS. There is an ongoing randomized clinical trial, REVIVE-TAVR,^13^ which will provide needed evidence.

### Limitations

There are several limitations to this study. First, this was a retrospective analysis of CMS data, introducing notable selection bias in procedural selection. Second, there is a lack of granular data with regard to the type or size of TAVR valves, imaging data, or laboratory values that preclude calculation of procedural risk. This also precludes determining types of valvular dysfunction. Lastly, high-quality analysis of post–TAVR balloon dilation and the TAVR plug procedure could not be performed.

## Conclusions

This represents a contemporary analysis of procedural incidence and volume following redo TAVR and TAVR explant. The annual volume of both redo TAVR and TAVR explant is increasing. TAVR reintervention beyond 5 years is predominantly performed via redo TAVR compared with TAVR explant. These results improve understanding of contemporary TAVR reintervention practices and heighten the importance of data-informed decision-making in the lifetime management of AS.

## Notes

Supplement 1. eTable 1. Patient and Operative Characteristics of Patients Undergoing Redo-TAVR and TAVR Explant eTable 2. ICD-9 and ICD-10 Codes Utilized for Data Collection eFigure 1. Flow Diagram of Cohort Identification and Group Creation eTable 3. Annual Volume of TAVR and TAVR Re-Interventions eFigure 2. Annual Volume of TAVR and TAVR Re-Intervention Procedures After Exclusion of Endocarditis Cases eTable 4. Annual Volume of TAVR and TAVR Re-Interventions After Exclusion of Endocarditis Cases eTable 5. Frequencies of Redo-TAVR and TAVR Explants at Different Time Intervals After Index TAVR eTable 6. Annual Volume of SAVR and SAVR Re-Interventions

Supplement 2. Data sharing statement
