# Supplementary material for: Contemporary Incidence and Procedural Volume of Transcatheter Aortic Valve Reintervention
Source: JAMA Cardiol. 2025 Sep 24;10(11):1201–6. doi: 10.1001/jamacardio.2025.3224 (PMC12461602; doi:10.1001/jamacardio.2025.3224)
Supplement: Supplement 1. — eTable 1. Patient and Operative Characteristics of Patients Undergoing Redo-TAVR and TAVR Explant eTable 2. ICD-9 and ICD-10 Codes Utilized for Data Collection eFigure 1. Flow Diagram of Cohort Identification and Group Creation eTable 3. Annual Volume of TAVR and TAVR Re-Interventions eFigure 2. Annual Volume of TAVR and TAVR Re-Intervention Procedures After Exclusion of Endocarditis Cases eTable 4. Annual Volume of TAVR and TAVR Re-Interventions After Exclusion of Endocarditis Cases eTable 5. Frequencies of Redo-TAVR and TAVR Explants at Different Time Intervals After Index TAVR eTable 6. Annual Volume of SAVR and SAVR Re-Interventions [file jamacardiol-e253224-s001.pdf]

Supplemental Online Content

Braasch MC, Pyeatte SR, He J, et al. Contemporary incidence and procedural volume of transcatheter aortic valve reintervention. *JAMA Cardiol*. Published online September 24, 2025. doi:10.1001/jamacardio.2025.3224

eTable 1. Patient and Operative Characteristics of Patients Undergoing Redo-TAVR and TAVR Explant

eTable 2. ICD-9 and ICD-10 Codes Utilized for Data Collection

eFigure 1. Flow Diagram of Cohort Identification and Group Creation

eTable 3. Annual Volume of TAVR and TAVR Re-Interventions

eFigure 2. Annual Volume of TAVR and TAVR Re-Intervention Procedures After Exclusion of Endocarditis Cases

eTable 4. Annual Volume of TAVR and TAVR Re-Interventions After Exclusion of Endocarditis Cases

eTable 5. Frequencies of Redo-TAVR and TAVR Explants at Different Time Intervals After Index TAVR

eTable 6. Annual Volume of SAVR and SAVR Re-Interventions

This supplemental material has been provided by the authors to give readers additional information about their work.

© 2025 Braasch et al. *JAMA Cardiol*.

eTable 1. Patient and Operative Characteristics of Patients Undergoing Redo-TAVR and TAVR Explant.

	**Redo-TAVR (n=2,374)**	**TAVR Explant (n=1,346)**
**Age, mean (SD), years**	80 [8]	74 [8]
**Male Sex**	1,263 (53.2%)	833 (61.9%)
**White Race**	2,177 (91.7%)	1,210 (89.9%)
**Diabetes Mellitus**	900 (37.9%)	509 (37.8%)
**Hypertension**	1,130 (47.6%)	641 (47.6%)
**Dyslipidemia**	1,410 (59.4%)	686 (51.0%)
**PVD**	541 (22.8%)	328 (24.4%)
**CVD**	234 (9.86%)	279 (20.7%)
**COPD**	741 (31.2%)	353 (26.2%)
**Previous MI**	181 (7.61%)	113 (8.40%)
**CHF**	2,061 (86.8%)	1,016 (75.5%)
**Atrial Fibrillation**	432 (18.2%)	365 (27.1%)
**CKD**	1,071 (45.1%)	481 (35.7%)
**Endocarditis**	50 (2.11%)	465 (34.5%)
**Dementia**	88 (3.71%)	21 (1.56%)
**Concomitant CABG**	N/a	217 (16.1%)
**Concomitant Mitral Valve Repair or Replacement**	N/a	336 (25.0%)
**Concomitant Thoracic Aortic Operation**	N/a	200 (14.9%)

Abbreviations: CABG: coronary artery bypass graft; CHF: congestive heart failure; CKD: chronic kidney disease; COPD: chronic obstructive pulmonary disease; CVD: cerebrovascular disease; MI: myocardial infarction; PVD: peripheral vascular disease; SD: standard deviation; TAVR: transcatheter aortic valve replacement.

© 2025 Braasch et al. *JAMA Cardiol*.

eTable 2. ICD-9 and ICD-10 Codes Utilized for Data Collection.

	ICD-9 and ICD-10 Codes
**SAVR**	ICD-9: 35.20, 35.21, 35.22<br>ICD-10: 02RF07Z, 02RF08Z, 02RF08N, 02RF0JZ, 02RF0KZ
**TAVR**	ICD-9: 35.05, 35.06<br>ICD-10: 02RF37Z, 02RF38Z, 02RF3JZ, 02RF3KZ, 02RF37H, 02RF38H, 02RF38N, 02RF3JH, 02RF3KH
**CABG**	ICD-9: 36.1x<br>ICD-10: 0210-0213
**Mitral valve repair or replacement**	ICD-9: 3502, 3512, 3522, 3523, 3524<br>ICD-10: 02QG0ZE, 02QG0ZZ, 02QG3ZE, 02QG3ZZ, 02QG4ZE, 02QG4ZZ, 02UG07Z, 02NG0ZZ, 027G04Z, 02QG0ZZ, 02UG08Z, 02UG0JZ, 02UG0KZ, 025G0ZZ, 027G0DZ, 027G0ZZ, 02BG0ZX, 02BG0ZZ, 02RG07Z, 02RG08Z, 02RG0JZ, 02RG0KZ, 02VG0ZZ, 02CG0ZZ, 02WG07Z, 02WG08Z, 02WG0JZ, 02WG0KZ
**Thoracic aortic operation**	ICD-9: 38.04, 38.14, 38.34, 38.45, 38.65, 38.85,<br>ICD-10: 02RW-02RX, 02QW-02QX, 027X
**Atrial fibrillation**	ICD-9: 427.3x<br>ICD-10: I48.91, I48.92
**Congestive heart failure**	ICD-9: 398.91, 402.01, 402.11, 402.91,404.01, 404.03, 404.11, 404.13,404.91, 404.93, 425.4-425.9, 428.x<br>ICD-10: I09.9,I11.0, I13.0, I13.2, I25.5, I42.0,I42.5-I42.9, I43.x, I50.x, P29.0
**Cerebrovascular disease**	ICD-9: 430.x-438.x<br>ICD-10: G45.x, G46.x, H34
**CKD**	ICD-9: 403.01, 403.11, 403.91, 404.02,404.03, 404.12, 404.13, 404.92,404.93, 582.x, 583.0-583.7, 585.x,586.x, 588.0, V42.0, V45.1, V56.x<br>ICD-10: I12.0, I13.1, N03.2-N03.7, N05.2-N05.7, N18.x, N19.x, N25.0, Z49.0-Z49.2, Z94.0, Z99.2
**COPD**	ICD-9: 416.8, 416.9, 490.x-505.x, 506.4, 508.1, 508.8<br>ICD-10: I27.8, I27.9, J40.x-J47.x, J60.x-J67.x, J68.4, J70.1, J70.3
**Dementia**	ICD-9: 290.x, 294.1, 331.2<br>ICD-10: F00.x-F03.x,F05.1, G30.x, G31.1
**Diabetes mellitus**	ICD-9: 250.0-250.3, 250.8, 250.9, 250.4-250.7<br>ICD-10: E10.0, E10.l, E10.6, E10.8, E10.9,E11.0, E11.1, E11.6, E11.8, E11.9,E12.0, E12.1, E12.6, E12.8, E12.9,E13.0, E13.1, E13.6, E13.8, E13.9,E14.0, E14.1, E14.6, E14.8,E14.9, E10.2-E10.5, E10.7, E11.2-E11.5,E11.7, E12.2-E12.5, E12.7,E13.2-E13.5, E13.7, E14.2-E14.5,E14.7
**Dyslipidemia**	ICD-9: 272.4<br>ICD-10: E78.4x, E78.5
**Endocarditis**	ICD-9: 036.42, 098.84, 112.81, 115.04, 115.14, 115.94, 421.0, 421.1, 421.9, 424.90, 424.91, 424.99<br>ICD-10: A32.82, A39.51, A52.03, A54.83, B33.21, B37.6, I01.1, I33.0, I33.9, I38, I39, M32.11
**Hypertension**	ICD-9: 401.x, 402.x<br>ICD-10: I10, I11.0, I11.9
**Previous MI**	ICD-9: 411, 411.1, 412<br>ICD-10:I24.1, I20.0, I25.2
**Peripheral vascular disease**	ICD-9: 093.0, 437.3, 440.x, 441.x,443.1-443.9, 447.1, 557.1,557.9, V43.4

© 2025 Braasch et al. *JAMA Cardiol*.

	ICD-10: I70.x, I71.x, I73.1, I73.8, I73.9, I77.1,I79.0, I79.2, K55.1, K55.8, K55.9, Z95.8, Z95.9, 093.0, 437.3, 440.x, 441.x,443.1-443.9, 447.1, 557.1,557.9, V43.4

Abbreviations: CABG: coronary artery bypass graft; CKD: chronic kidney disease; COPD: chronic obstructive pulmonary disease; ICD: International Classification of Diseases; MI: myocardial infarction; SAVR: surgical aortic valve replacement; TAVR: transcatheter aortic valve replacement.

© 2025 Braasch et al. *JAMA Cardiol*.

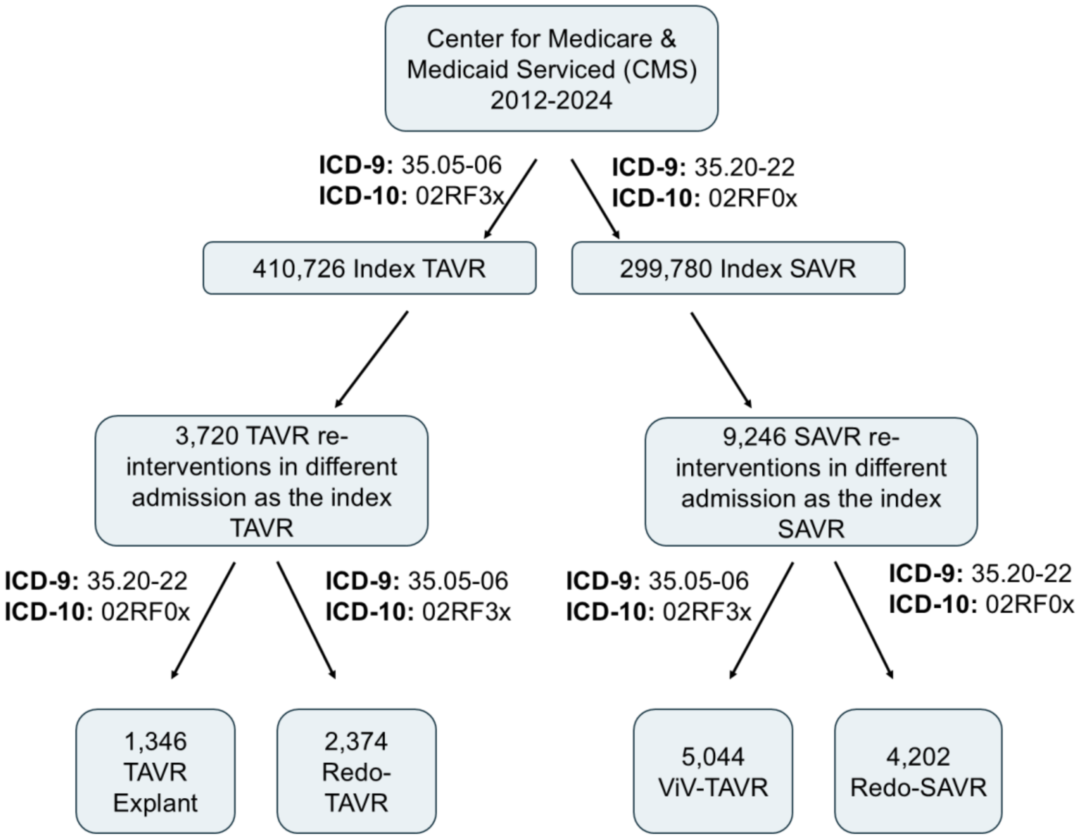

eFigure 1. Flow Diagram of Cohort Identification and Group Creation.

Abbreviations CMS: Center for Medicare & Medicaid Services; ICD: International Classification of Diseases; SAVR: surgical aortic valve replacement; TAVR: transcatheter aortic valve replacement; ViV-TAVR: valve-in-valve transcatheter aortic valve replacement.

eTable 3. Annual Volume of TAVR and TAVR Re-Interventions.

	TAVR	TAVR at Risk of Re-Intervention	Redo-TAVR	TAVR Explant	Total Annual TAVR Re-Interventions	Annual Re-Intervention Incidence
**2012[a]**	5496	5496	N/a	N/a	43	0.78%
**2013[a]**	9371	14033	N/a	N/a	64	0.46%
**2014**	14056	26059	80	23	103	0.40%
**2015**	19889	42486	118	29	147	0.35%
**2016**	28087	65036	113	34	147	0.23%
**2017**	34888	92056	115	64	179	0.19%
**2018**	37315	128256	169	85	254	0.20%
**2019**	44620	158513	176	91	267	0.17%
**2020**	44319	185513	208	158	366	0.20%
**2021**	48471	211076	265	207	472	0.22%
**2022**	49372	234890	315	223	538	0.23%
**2023**	49642	256487	446	268	714	0.28%
**2024[b]**	25200 (50400)	N/a	284 (568)	142 (284)	426 (852)	N/a

[a]Data censored with tabulations <11 per Healthcare Cost & Utilization Project Data Use Agreement for patient confidentiality. [b]Data for 2024 presented as 6 months of reported data with annualized data in parentheses, and no number at risk calculable with lack of full year of data. Abbreviation: TAVR: transcatheter aortic valve replacement

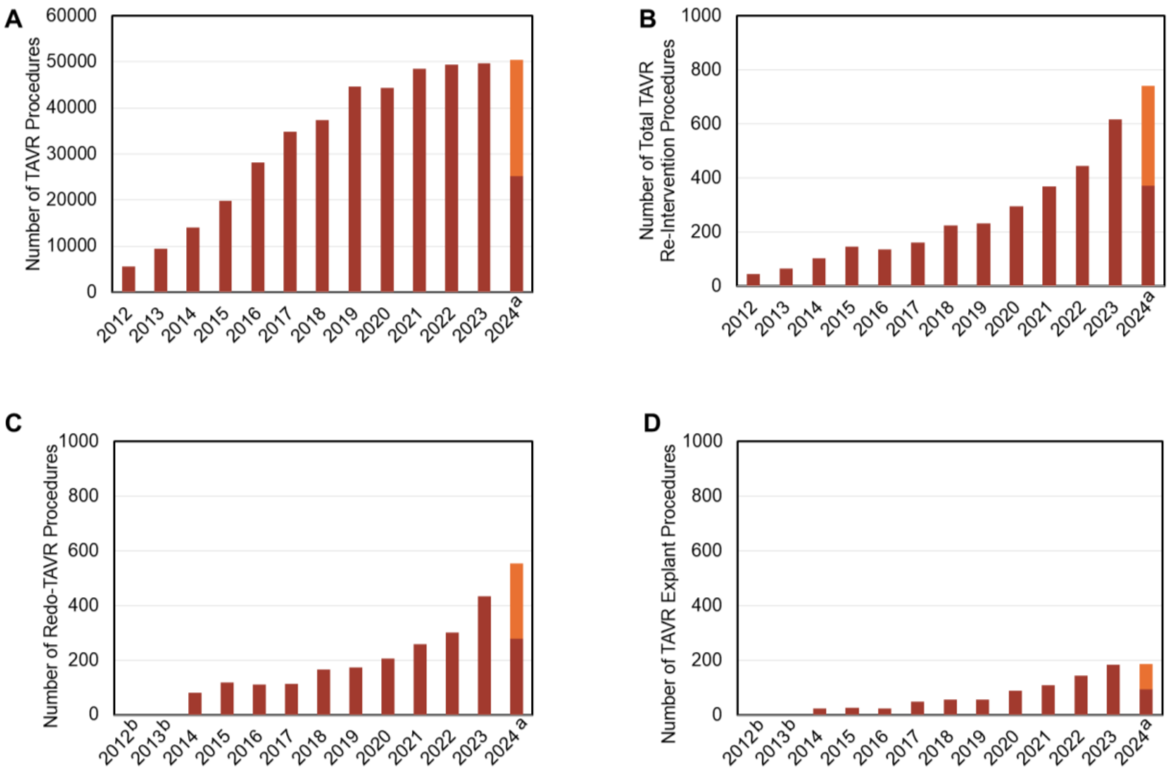

eFigure 2. Annual Volume of TAVR and TAVR Re-Intervention Procedures After Exclusion of Endocarditis Cases.

Figure 1 Legend: A) Number of TAVR procedures performed per year. The Y-axis scale for Panel A differs from the y-axis scales for Panels B-D. B) Total number of TAVR re-intervention procedures performed per year. C) Number of Redo-TAVR procedures performed per year. D) Number of TAVR Explant procedures performed per year. [a]Procedural volume for 2024 annualized (orange bar) from 6 months of available data (red bar). [b]Data censored with results <11 per Healthcare Cost & Utilization Project Data Use Agreement for patient confidentiality. Abbreviation: TAVR: transcatheter aortic valve replacement.

eTable 4. Annual Volume of TAVR and TAVR Re-Interventions After Exclusion of Endocarditis Cases.

	TAVR	Redo-TAVR	TAVR Explant	Total Annual TAVR Re-Interventions
**2012[a]**	5496	N/a	N/a	43
**2013[a]**	9371	N/a	N/a	64
**2014**	14056	80	23	103
**2015**	19889	118	27	145
**2016**	28087	111	25	136
**2017**	34888	114	48	162
**2018**	37315	166	57	223
**2019**	44620	174	57	231
**2020**	44319	206	90	296
**2021**	48471	259	110	369
**2022**	49372	301	144	445
**2023**	49642	433	185	618
**2024[b]**	25200 (50400)	277 (554)	93 (186)	370 (740)

[a]Data censored with tabulations <11 per Healthcare Cost & Utilization Project Data Use Agreement for patient confidentiality. [b]Data for 2024 presented as 6 months of reported data with annualized data in parentheses. Abbreviation: TAVR: transcatheter aortic valve replacement

eTable 5. Frequencies of Redo-TAVR and TAVR Explants at Different Time Intervals After Index TAVR.

	All Re-Interventions (n=3,720)	All Redo-TAVRs (n=2,374)	All TAVR Explants (n=1,346)	TAVR Explants in Patients Diagnosed with Endocarditis (n=465)	TAVR Explants in Patients not Diagnosed with Endocarditis (n=881)
**<3 months**	600	410	190	35	155
**3-6 months**	314	175	139	72	67
**6 months – 12 months**	422	169	253	132	121
**1-2 years**	496	237	259	108	151
**2-3 years**	356	180	176	60	116
**3-4 years**	364	215	149	42	107
**4-5 years**	349	263	86	16	70
**5-6 years[a]**	330	280	50	N/a	N/a
**6-7 years[a]**	240	208	32	N/a	N/a
**7-8 years[a]**	129	122	N/a	N/a	N/a
**8-9 years[a]**	75	72	N/a	N/a	N/a
**9-10 years[a]**	28	28	N/a	N/a	N/a
**>10 years[a]**	17	15	N/a	N/a	N/a

[a]Data censored with tabulations <11 per Healthcare Cost & Utilization Project Data Use Agreement for patient confidentiality.

Abbreviation: TAVR: transcatheter aortic valve replacement.

eTable 6. Annual Volume of SAVR and SAVR Re-Interventions.

	SAVR	SAVR at Risk of Re-Intervention	ViV-TAVR	Redo-SAVR	Total Annual SAVR Re-Interventions	Annual Re-Intervention Incidence
**2012[a]**	35233	35233	N/a	N/a	86	0.24%
**2013[a]**	35888	68269	N/a	N/a	207	0.30%
**2014[a]**	34283	98174	N/a	N/a	232	0.24%
**2015**	32650	125016	52	302	354	0.28%
**2016**	29460	147313	115	339	454	0.31%
**2017**	25437	164334	205	376	581	0.35%
**2018**	23253	177811	337	416	753	0.42%
**2019**	19449	186057	513	436	949	0.51%
**2020**	14101	187814	616	421	1037	0.55%
**2021**	14568	187593	782	420	1202	0.64%
**2022**	14280	186609	931	409	1340	0.72%
**2023**	14268	185523	958	397	1355	0.73%
**2024[b]**	6910 (13820)	N/a	518 (1036)	178 (356)	696 (1392)	N/a

[a]Data censored with tabulations <11 per Healthcare Cost & Utilization Project Data Use Agreement for patient confidentiality. [b]Data for 2024 presented as 6 months of reported data with annualized data in parentheses, and no number at risk calculable with lack of full year of data. Abbreviation: SAVR: surgical aortic valve replacement; ViV-TAVR: valve-in-valve transcatheter aortic valve replacement.
