# Supplementary material for: Contemporary Incidence and Procedural Volume of Transcatheter Aortic Valve Reintervention
Source: JAMA Cardiol. 2025 Sep 24;10(11):1201–6. doi: 10.1001/jamacardio.2025.3224 (PMC12461602; doi:10.1001/jamacardio.2025.3224)
Supplement: Supplement 2. — Data sharing statement [file jamacardiol-e253224-s002.pdf]

Data Sharing Statement

Braasch. Contemporary Incidence and Procedural Volume of Transcatheter Aortic Valve Reintervention. *JAMA Cardiol*. Published September 24, 2025.
doi:10.1001/jamacardio.2025.3224

Data
Data available: No

Additional Information
Explanation for why data not available: CMS data use agreement precludes data sharing
